# Effects of Diets Supplemented with Branched-Chain Amino Acids on the Performance and Fatigue Mechanisms of Rats Submitted to Prolonged Physical Exercise

**DOI:** 10.3390/nu4111767

**Published:** 2012-11-16

**Authors:** Gina Falavigna, Jonas Alves de Araújo Junior, Marcelo Macedo Rogero, Ivanir Santana de Oliveira Pires, Rogério Graça Pedrosa, Eivor Martins Junior, Inar Alves de Castro, Julio Tirapegui

**Affiliations:** 1 Department of Food and Experimental Nutrition, Faculty of Pharmaceutical Sciences, University of São Paulo, São Paulo 05508900, Brazil; Email: ginafalavigna@yahoo.com.br (G.F.); xonasxr@hotmail.com (J.A.A.J.); ivanirpires@yahoo.com.br (I.S.O.P.); rogergp28@gmail.com (R.G.P.); inarcastro@gmail.com (I.A.C.); tirapegu@usp.br (J.T.); 2 Department of Nutrition, School of Public Health, University of São Paulo, São Paulo 01246904, Brazil; 3 Department of Physiology and Biophysics, Institute of Biomedical Sciences, University of São Paulo, São Paulo 05508900, Brazil; Email: eivor@zipmail.com.br

**Keywords:** amino acids, dietary supplements, exercise, fatigue, sports performance

## Abstract

This study aimed to determine the effects of diets chronically supplemented with branched-chain amino acids (BCAA) on the fatigue mechanisms of trained rats. Thirty-six adult Wistar rats were trained for six weeks. The training protocol consisted of bouts of swimming exercise (one hour a day, five times a week, for six weeks). The animals received a control diet (C) (*n* = 12), a diet supplemented with 3.57% BCAA (S1) (*n* = 12), or a diet supplemented with 4.76% BCAA (S2) (*n* = 12). On the last day of the training protocol, half the animals in each group were sacrificed after one hour of swimming (1H), and the other half after a swimming exhaustion test (EX). Swimming time until exhaustion was increased by 37% in group S1 and reduced by 43% in group S2 compared to group C. Results indicate that the S1 diet had a beneficial effect on performance by sparing glycogen in the soleus muscle (*p* < 0.05) and by inducing a lower concentration of plasma ammonia, whereas the S2 diet had a negative effect on performance due to hyperammonemia (*p* < 0.05). The hypothalamic concentration of serotonin was not significantly different between the 1H and EX conditions. In conclusion, chronic BCAA supplementation led to increased performance in rats subjected to a swimming test to exhaustion. However, this is a dose-dependent effect, since chronic ingestion of elevated quantities of BCAA led to a reduction in performance.

## 1. Introduction

Fatigue may be defined as the inability to maintain the expected muscle strength, leading to a reduced performance during prolonged exercise. The mechanisms that directly affect the muscles are peripheral and those residing in the brain are central [1,2]. The following events are considered to be the five major metabolic causes of fatigue: (1) muscle phosphocreatinine depletion; (2) proton accumulation in muscle (acidosis); (3) muscle glycogen depletion; (4) reduced blood glucose concentration; (5) increased ratio of specific amino acids in plasma (free tryptophan/branched-chain amino acids (TRP_L_/BCAA)). The first three causes are directly related to peripheral fatigue and the last two are probably related to central fatigue [1,3,4]. In addition to these causes, the increased plasma ammonia concentration that occurs during prolonged exercise can be highly toxic and is associated with both peripheral and central fatigue [5,6,7]. 

In contrast to the liver, which can oxidize the twenty amino acids present in the tissue protein of the organism, skeletal muscle can oxidize only six: BCAA, aspartate, asparagine and glutamate [8,9]. In muscle tissue, BCAA participates in protein synthesis and act as a precursor in the synthesis of other amino acids, in addition to being used as energy substrates during physical exercise [10,11,12]. Of the three BCAAs, leucine is the one primarily responsible for the stimulation of protein synthesis induced by intake of a mixed meal. The stimulatory effect of leucine on protein synthesis is mediated through upregulation of the initiation of mRNA translation [13]. 

The hypothesis of central fatigue suggests that the increase in serotonin or 5-hydroxytryptamine (5-HT) concentration in the brain during prolonged exercise may be directly related to the development of fatigue and to the consequent reduction in performance. On the other hand, BCAA supplementation may act on the reduction of the plasma TRP_L_/BCAA ratio since these amino acids have the same mechanism of transport through the blood-brain barrier, a fact that may reduce the influx of TRP_L_ (a serotonin precursor) in the central nervous system (CNS). This occurrence may cause a reduced 5-HT synthesis in the brain, consequently delaying the symptoms of fatigue [3,7,14,15]. In addition, BCAA may act on glycogen metabolism during prolonged exercise, since supplementation with these amino acids can preserve hepatic and muscle glycogen possibly resulting in increased performance [16,17].

The effects of BCAA supplementation on performance in prolonged exercise have not been clearly demonstrated in the literature, since there are reports of both beneficial effects [18,19,20] and of a non-ergogenic effect [20,21]. However, these controversial results may be justified by differences between experimental protocols, such as an animal or human models; quantity, time of supply (chronic or acute) and route of administration of the supplement: type, duration and intensity of exercise.

In many of the animal studies, in which the effects of BCAA supplementation on metabolism and physical performance were determined, a single BCAA dose (acute form) was administered before exercise, by gavage [22] or by intraperitoneal injection [23,24]. We hypothesized that chronic BCAA supplementation (through the diet, using different BCAA concentrations) would increase performance in rats subjected to a swimming exhaustion test. Furthermore, we hypothesized that the mechanism by which chronic BCAA supplementation improved the performance is via preservation of liver and muscle glycogen stores or decreasing the 5-HT concentration in the hypothalamus. 

## 2. Experimental Section

### 2.1. Animals

The study was conducted on male Wistar rats weighing on average 240 ± 18 g at the beginning of the experiment, supplied by the animal house of the Faculty of Pharmaceutical Sciences, University of São Paulo. The animals were kept in individual cages in a room with controlled temperature of 22 ± 2 °C, on an inverted 12 h light/12 h dark cycle, with lights on at 19:00 h. 

The animals had free access to food and water and the consumption of ration and body weight was determined three times a week. The final weight was measured on the day of sacrifice. The animals were followed for a total period of seven weeks, with the first week being devoted to adaptation to the environment and to the diet, and the remaining weeks being used for physical training. 

All procedures carried out on the rats were approved by the Ethics Committee on Animal Experimentation of the Faculty of Pharmaceutical Sciences, University of São Paulo, according to the guidelines of the Brazilian College on Animal Experimentation.

### 2.2. Experimental Diets

The animals received a control (C), supplemented 1 (S1) or supplemented 2 (S2) diet throughout the experiment. The C diet was elaborated according to the recommendations of the American Institute of Nutrition (AIN-93M) for the maintenance of adult rodents [25] and the S1 and S2 diets were elaborated according to the AIN-93M and respectively supplemented with 3.57% and 4.76% BCAA. The diets supplemented with 3.57% and 4.76% BCAA represent an increase of 50% and 100% of BCAA quantity in comparison to the amount of these amino acids in the C diet, respectively. The amino acids were supplied by Ajinomoto Interamericana Indústria e Comércio Ltda (São Paulo, Brazil).

In the formulation of the supplemented rations the amount of starch equivalent to the addition of BCCA (in grams) was removed so that the diets would be isocaloric with the non-supplemented ration. Table 1 presents the formulation of the three diets, which were elaborated according to the AIN-93M. 

**Table 1:** Formulation of the experimental diets * (g/kg diet).

Ingredients	C **	S1 **	S2 **
Starch	620.692	584.992	573.092
Casein (>85% protein)	140.000	140.000	140.000
Saccharose	100.000	100.000	100.000
Soy oil	40.000	40.000	40.000
Fiber (cellulose)	50.000	50.000	50.000
Mineral mix	35.000	35.000	35.000
Vitamin mix	10.000	10.000	10.000
l-Cystine	1.800	1.800	1.800
Choline bitartrate (41.1% de colina)	2.500	2.500	2.500
Tert-butylhydroquinone	0.008	0.008	0.008
l-Isoleucine	- ***	8.85	11.8
l-Leucine l	- ***	16.35	21.8
l-Valine	- ***	10.5	14.0
BCAA total addition	-	35.7	47.6

* The C diet was elaborated according to the recommendations of the American Institute of Nutrition (AIN-93M) for the maintenance of adult rodents [25] and the S1 and S2 diets were elaborated according to the AIN-93M and respectively supplemented with 3.57% and 4.76% BCAA; ** (control diet (C), supplemented 1 diet (S1) or supplemented 2 diet (S2)); *** Estimated branched-chain amino acids (BCAA) composition of the AIN-93M diet (g/kg diet): l-isoleucine: 5.9; l-leucine: 10.9; l-valine: 7.0.

### 2.3. Training Protocol

A swimming system developed by Vieira was used [26]. The training protocol lasted six weeks; in the first two weeks the animals were adapted to the water medium and exercised with increasing overloads attached to the tail until an overload corresponding to 5% of total body weight was reached. This final overload was used until the end of the training protocol, which was performed five times a week, for one hour a day, between 9:00 and 12:00, with a water temperature of 32 °C [27]. The overloads were corrected weekly according to the variations in animal weight. The efficiency of the training protocol was assessed on the basis of maximum activity of the enzyme citrate synthase in the soleus muscle, with a group of sedentary animals being used as the control for this parameter.

### 2.4. Animal Sacrifice and Sample Collection

On the last day of the protocol the animals were deprived of food for two hours before the beginning of physical activity and then decapitated under two conditions; after one hour of swimming (1H) and after the swimming exhaustion test (EX). The 1H groups were sacrificed between 9:00 and 11:00 and swimming time was recorded individually for the animals in the EX groups. Time of swimming exhaustion test was defined as the time when the animal remained submersed for approximately 12 s [27]. We have chosen to kill the animals after one hour of swimming because this time point allowed investigating the effects of BBCA supplementation in a similar situation to the daily protocol training. On the other hand, the results from the animals killed after the swimming exhaustion test allowed evaluating the effect of BCAA supplementation on the peripheral fatigue and central fatigue mechanisms.

Immediately before sacrifice, 25 μL blood was directly collected into heparinized capillary tubes from the tip of the animals’ tail for lactate determination. After decapitation, blood was collected and centrifuged in order to obtain the plasma and serum fractions. The hypothalamus, liver and gastrocnemius and soleus muscles were removed, weighed, wrapped and immediately frozen in liquid nitrogen. The samples were stored in a freezer at −80 °C for later biochemical analysis.

### 2.5. Biochemical Analyses

The concentrations of the following substances were determined: blood lactate (by an electrochemical technique after sample stabilization with 2% sodium fluoride using a 1500 lactimeter model from Yellow Springs Instruments, Yellow Springs, OH, USA), plasma glucose (with a Labtest Diagnóstica kit, Glucose PAP Liquiform, Lagoa Santa, Minas Gerais, Brazil), plasma ammonia (with a Sigma Diagnostics kit, St. Louis, MO, USA), serum insulin (by radioimmunoassays using at Biotrak™ kit, Amersham International, Piscataway, NJ, USA), muscle glycogen—gastrocnemius and soleus—and liver glycogen [24], muscle protein—gastrocnemius—and liver protein [28], maximum citrate synthase activity [29]. 

Hypothalamic serotonin was determined by HPLC with electrochemical detection, according to the method of Ribeiro [30] and modified from Cipolla-Neto [31]. The chromatographic system (Shimadzu, Kyoto, Japan) composed by an isocratic LC-10AD *vp* HPLC pump, a Resolve 5 μm spherical C18, 3.9 × 150 mm steel column, and a L-ECD-6A electrochemical detector operated in DC mode, was controlled by the Shimadzu CLASS-VP Software through a System Interface Module. Each hypothalamus was sonicated (microson XL 2005, Heat System Inc., Farmingdale, NY, USA) for 10 s in 120 μL of ice cold 0.1 M perchloric acid containing 0.02% EDTA and 0.02% sodium bisulfite. Protein and cell debris were removed by centrifugation (14,000× *g*, 2 min) (Eppendorf 5415C centrifuge, Brinkmann Instruments Inc., Westbury, NY, USA). The clear supernatant (60 μL) was injected into the system through a syringe loading injector (20 μL loop, Mod 7125, Rheodyne Inc., Oak Harbor, WA, USA). The chromatographic system was operated with the following phase at 30 °C: 0.1 M sodium acetate, 0.1 M citric acid, 0.15 M EDTA, 35% methanol, pH 3.7 at a constant flow rate of 1.0 mL/min. The detector potential was adjusted to a steady value of +900 mV (*vs.* Ag/AgCl reference electrode). The total run was 15 min and, typically, serotonin was eluted at 10 min. Free fatty acids were determined by the method of Regouw [32] and samples were read in a spectrophotometer.

### 2.6. Statistical Methods

Results were expressed as mean ± S.D. Variance homogeneity was tested by the Hartley test. All data were considered statistically significant when the probability of Type I error was <0.05. The effect of supplementation on body weight, diet and BCAA intake was evaluated after one hour of swimming (1H) and also after the swimming exhaustion test (EX) by one way ANOVA followed by the Tukey HSD test. When variance homogeneity was not observed, data were evaluated by the Kruskal-Wallis ANOVA test. Except for two variables (liver and gastrocnemius muscle glycogen concentrations) that did not show variance homogeneity, mixed design factorial ANOVA was applied to analyze the biochemical variables considering as independent factors the supplementation at three levels (−1 = AIN-93 M, 0 = AIN-93 M + 3.57% BCAA and +1 = AIN-93 M + 4.76% BCAA), and the time of exercise before the sacrifice at two levels (−1 = 1 h and +1 = exhaustion). All calculations and graphs were made using the Statistica 7.1 software [33] (StatSoft Inc., Tulsa, OK, USA). 

## 3. Results

Daily food intake did not differ significantly between the experimental groups studied, whereas daily BCAA ingestion was significantly higher in the groups supplemented with BCAA (Table 2). There was no difference in final body weight between animals receiving the various diets.

**Table 2:** Initial and final body weight (g), daily food intake (g/day) and estimated BCAA ingestion (mg/day) of experimental groups trained for 1 h (1H) or trained and submitted to exhaustion (EX), in animals that received a control, supplemented 1 (S1) or supplemented 2 (S2) diet.

Variables ^1^	*Control*		*S1*		*S2*	
	1H	EX	1H	EX	1H	EX
Initial body weight (g)	238.9 ± 17.1	240.5 ± 14.4	242.5 ± 7.4	240.8 ± 11.9	235.9 ± 29.9	236.07 ± 26.5
Final body weight (g)	336.6 ± 9.9	337.6 ± 27.4	340.5 ± 2.5	340.5 ± 21.5	336.2 ± 20.9	337.0 ± 22.9
Diet intake (g/day)	21.6 ± 1.1	21.1 ± 0.9	21.6 ± 1.0	20.7 ± 2.0	20.7 ± 1.6	21.5 ± 1.5
BCAA (mg/day)	515.5 ± 26.8 ^a^	502.2 ± 22.4 ^a^	769.9 ± 35.3 ^b^	738.6 ± 70.4 ^b^	985.3 ± 74.8 ^c^	1022.8 ± 70.5 ^c^

^1^ Values are expressed as mean ± SD (*n* = 6). Probability value obtained by one way ANOVA followed by the Tukey HSD test. Values followed by the same superscript letters (a, b, c) are not statistically different (*p* < 0.05).

**Figure 1:**
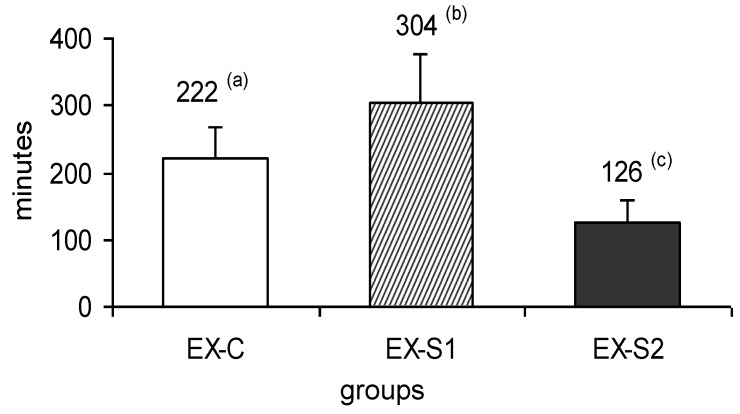
Swimming time (minutes) of the experimental groups submitted to exhaustion (EX) that received a control diet (C), supplemented 1 diet (S1) or supplemented 2 diet (S2). Columns with different letters are statistically different (*p* < 0.05, one way ANOVA followed by the Tukey HSD test). Values are expressed as mean ± S.D. (*n* = 6).

A significant 75% increase in maximum citrate synthase activity (soleus muscle) was observed in the trained groups (1H: 5.84 ± 1.33 and EX: 5.95 ± 1.05 μmol/min/g fresh tissue) compared to the sedentary group (3.37 ± 0.73 μmol/min/g fresh tissue). As noted in Figure 1. 

Swimming time for the EX-S1 group was significantly higher (37%) than the EX-C group. On the other hand, the EX-S2 group showed a significant decrease in performance of 43% and 59% in relation to the EX-C and EX-S1 groups, respectively.

**Figure 2:**
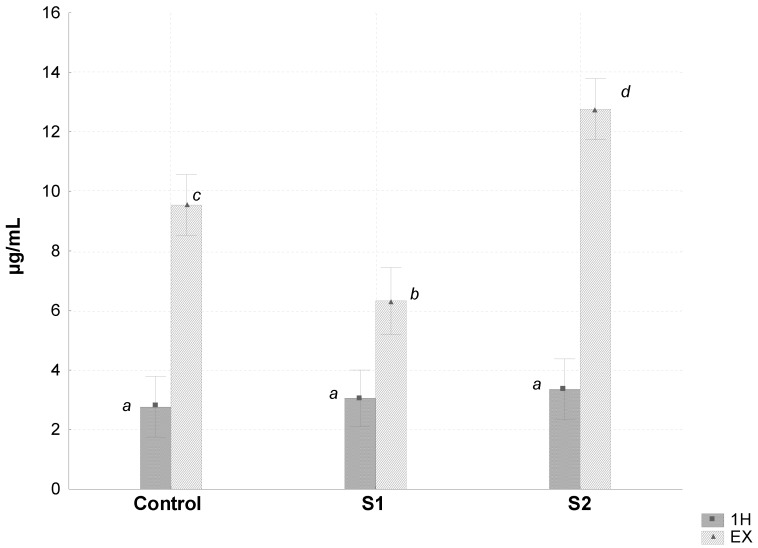
Plasma ammonia in the experimental groups trained for 1 h (1H) or trained and submitted to exhaustion (EX), in animals that received a control, supplemented 1 (S1) or supplemented 2 (S2) diet. Columns with different letters are statistically different (*p* < 0.05, probability value obtained by Factorial ANOVA for the factors interaction “supplementation × time of exercise before the sacrifice”). Values are expressed as mean ± SD (*n* = 6).

EX-S2 group demonstrated a significantly greater plasma concentration of ammonia than those of the EX-C and EX-1H groups, while the EX-S1 group presented a significant 34% decrease, compared to the EX-C group (Figure 2). The EX-S1 group showed a significantly greater soleus muscle glycogen concentration than that of the EX-C (Table 3). 

**Table 3:** Determined parameters in blood and tissue muscle of experimental groups trained for 1 h (1H) or trained and submitted to exhaustion (EX), in animals that received a control, supplemented 1 (S1) or supplemented 2 (S2) diet.

Variables ^1^	Control		S1		S2		*p* ^2^	*p* ^3^	*p* ^4^
	1H	EX	1H	EX	1H	EX			
Plasma glucose (mg/dL)	144.9 ^a^ ± 34.2	55.6 ^b^ ± 14.0	148.2 ^a^ ± 12.5	60.8 ^b^ ± 13.2	144.0 ^a^± 15.2	55.0 ^b^ ± 12.9	0.962	<0.001	-
Plasma free fatty acids (mmol/L)	0.27 ± 0.05	0.26 ± 0.04	0.29 ± 0.07	0.27 ± 0.05	0.25 ± 0.04	0.26 ± 0.04	0.525	0.669	-
Serum insulin (ng/dL)	3.03 ^b,c^ ± 0.73	1.48 ^a^ ± 0.30	2.89 ^b,c^ ± 0.53	1.43 ^a^ ± 0.28	3.58 ^c^ ± 0.35	2.14 ^a,b^ ± 0.27	0.002	<0.001	-
Blood lactate (mmol/L)	14.37 ^a^ ± 1.31	18.99 ^b^ ± 2.59	13.97 ^a^ ± 1.84	18.64 ^b^ ± 5.04	16.02 ^a^ ± 3.19	19.21 ^b^ ± 4.80	0.742	<0.001	-
Liver glycogen (mg/100 mg tissue)	0.76 ^a^ ± 0.16	0.10 ^b^ ± 0.02	0.72 ^a^ ± 0.21	0.33 ^b^ ± 0.04	0.79 ^a^ ± 0.15	0.32 ^b^ ± 0.03 ^b^	-	-	<0.001
Gastrocnemius muscle glycogen (mg/100 mg tissue)	0.20 ^a^ ± 0.06	0.11 ^b^ ± 0.03	0.24 ^a^ ± 0.06	0.13 ^b^ ± 0.03	0.21 ^a^ ± 0.05	0.10 ^b^ ± 0.02	-	-	<0.001
Soleus muscle glycogen (mg/100 mg tissue)	0.33 ^a,b^ ± 0.08	0.23 ^c^ ± 0.05	0.41 ^b^ ± 0.02	0.33 ^a,d^ ± 0.05	0.34 ^a,b^ ± 0.03	0.24 ^c,d^ ± 0.03	<0.001	<0.001	-
Hypothalamic serotonin (pg/mg tissue)	242.3 ^a^ ± 59.6	330.5 ^a,b^ ± 74.5	256.1 ^a,b^ ± 52.2	321.8 ^a,b^ ± 94.9	272.8 ^a,b^ ± 44.9	384.6 ^b^ ± 108.6	0.359	0.011	-

^1^ Values are expressed as mean ± SD (*n* = 6) and there were no significant interactions (*p* > 0.05); ^2^ Probability value obtained by Factorial ANOVA to the factor “supplementation”; ^3^ Probability value obtained by Factorial ANOVA to the factor “time of exercise before the sacrifice”; ^4^ Probability value obtained by Kruskal-Wallis ANOVA; Means of each parameter followed by the same letter (^a^, ^b^, ^c^, ^d^) did not differ significantly between groups (*p* < 0.05).

## 4. Discussion

Food intake did not differ between groups, indicating that the supplementation of BCAA did not change the rats’ food consumption. Similarly, Shimomura [16] and Araujo, Jr. [34] who also administered BCAA to rats through diet, found no differences between the groups in relation to food intake. In addition, there were no statistically significant differences in the final weights between groups (338 ± 17 g), indicating that the supplemented diets (S1 and S2) had no effect on the final weight of the animals. 

The improvement in the animals’ physical condition was demonstrated by the 75% increase in maximum citrate synthase activity in trained groups, when compared to the sedentary group. This demonstrated that the training protocol was effective in increasing the oxidative metabolism in the trained animals [25,35]. At the same time, no significant differences in maximum citrate synthase activity were observed between trained groups, indicating that the physical condition of the animals submitted to the training protocol was similar. Thus, the differences between the trained groups (1H and EX) in the present study were exclusively due to the effect of BCAA supplementation.

Results show that BCAA improved performance in the group fed on the diet supplemented with 3.57% BCAA (S1); this group showed a 37% increase in time to exhaustion compared to the group receiving the C diet. On the other hand, the group receiving the S2 diet supplemented with 4.75% BCAA showed a significant decrease in swimming time, when compared to the other groups. Among the possible hypotheses related to this result, we highlight the availability of plasma glucose and liver and muscle glycogen, which are relevant substrates for supplying energy during exercise of moderate intensity and long duration. During prolonged exercise to exhaustion, there is increased flow through the glycolytic pathway, resulting in decreased glucose levels and the concomitant increase in plasma lactate concentration, which are important factors in the etiology of fatigue during exercise [3,9]. In this study, the groups submitted to exhaustion had reduced blood glucose and liver and gastrocnemius muscle glycogen concentrations, with a concomitant increase in plasma lactate concentration compared to their respective control groups when exercised for one hour. This finding demonstrates that the effects of chronic supplementation with BCAA on performance in trained rats do not involve changes in glucose metabolism.

Ammonia is a ubiquitous metabolic product producing multiple effects on physiological and biochemical systems. Its concentration in several body compartments is elevated during exercise, predominantly by the increased activity of the purine nucleotide cycle in skeletal muscle [12,36]. Depending on the intensity and duration of exercise, muscle ammonia may be elevated to the extent that it leaks (diffuses) from muscle to blood, and thereby can be carried to other organs [12]. The direction of movement of ammonia or the ammonium ion is dependent on concentration and pH gradients between tissues. As such, ammonia can also cross the blood-brain barrier, although the rate of diffusion of ammonia from blood to brain during exercise is unknown [37,38]. It seems reasonable to assume that exhaustive exercise may induce a state of acute ammonia toxicity which, although transient and reversible relative to disease states, may be severe enough in critical regions of the central nervous system (CNS) to affect continuing coordinated activity. Regional differences in brain ammonia content, detoxification capacity, and specific sensitivity may account for the variability of precipitating factors and latency of response in CNS-mediated dysfunction arising from an exercise stimulus, e.g., motor incoordination, ataxia and stupor. There have been numerous suggestions that elevated ammonia is associated with, or perhaps is responsible for, exercise fatigue, although evidence for this relies extensively on temporal relationships [3,9]. 

In our study, the greater chronic ingestion of BCAA, through the diet, in the EX-S2 group, increased the plasma concentration of ammonia in this group [27,33], which might have contributed to the reduction time of exercise tolerance in the EX-S2 group in relation to other groups submitted to the exhaustion test. This fact suggests that diets featuring high doses of chronically-administered BCAA may be toxic and lead to early fatigue during prolonged exercise. According to Shimomura [16], chronic BCAA supplementation is effective in increasing plasma BCAA concentration in a resting situation because the activity of hepatic branched-chain amino acid transaminase (BCAAT) is very low [33,39], in turn increasing the availability of BCAA to the blood circulation and, subsequently, to the skeletal musculature. In muscle tissue, BCAA are transaminated with α-ketoglutarate in a reaction catalyzed by BCAA aminotransferase, which generates one branched-chain α-keto acid (BCKA) and glutamate. Oxidative decarboxylation of BCKA in muscle is low since the activity of the branched-chain α-keto acid dehydrogenase (BCKAD) complex is reduced in this tissue. BCKA are therefore released into the circulation for subsequent metabolism in the liver, where the activity of the hepatic BCKAD complex is high [15]. This mechanism is probably associated with an increase in hepatic BCAAT activity, which seems to be proportional to the ingested amount of BCAA [16,33]. Therefore, chronic ingestion of large amounts of BCAA increases hepatic BCAAT activity, favoring the formation of BCKA in the liver itself. This, in turn, can increase hepatic BCAA catabolism, probably favoring during the swimming test to exhaustion, which may have increased the ammonia concentration in the EX-S2 group in relation to the other groups. 

Central fatigue is related to the increased release of neurotransmitters, particularly 5-hydroxytryptamine (serotonin). This hypothesis stems from the fact that exhaustive exercise results in a gradual increase in the concentration of plasma free fatty acids, which compete with tryptophan for binding to plasma protein albumin [11,40]. Thus, there is an increased concentration of free tryptophan, through the displacement of this amino acid from plasma albumin. Under normal physiological conditions, tryptophan circulates in the blood with the main fraction (70%–90%) bound to plasma albumin. BCAAs compete with free tryptophan by binding to the same transport of neutral amino acids in the blood-brain barrier. Thus, the entry of tryptophan into the central nervous system (CNS) is regulated by the ratio plasma free tryptophan: BCAA and favored by the decrease in BCAA concentration in the blood, resulting from the increased rate of oxidation [41]. Thus, the decrease in glycogen stores, increased oxidation of BCAA and high concentration of plasma fatty acids act as important factors in increasing the synthesis of the neurotransmitter serotonin in the CNS, and this is dependent on the availability of tryptophan—a precursor of serotonin—in the CNS. The increased synthesis of serotonin during exercise may be related to the development of central fatigue, because this neurotransmitter has several physiological functions, since it operates by mood, lethargy, individual behavior, regulation of sleep, body temperature and blood pressure, appetite suppression and changes in perceived exertion [41]. In the present study, measurement of hypothalamic serotonin concentrations and plasma free fatty acids demonstrated no significant differences between the dietary groups, indicating that chronic BCAA supplementation was not effective in improving the main parameters indicative of central fatigue.

From the results of our study, it can be suggested that there is a tolerance level of BCAA ingestion in rats submitted to prolonged physical exercise. It should be noted that acute and sub-acute toxicity studies of BCAAs using mice and rats and a chronic toxicity study using rats have been reported. In these studies, the BCAA composition used was a 2.1:1:1.2 leucine:isoleucine:valine ratio, and no animals died from the single dose of 10 g of BCAA/kg body weight in the acute toxicity study, and the half-maximal lethal dose was estimated as >10 g/kg body weight. No toxic effects of BCAA were observed at a dose of 2.5 g/kg body weight per day for three months or 1.25 g/kg body weight per day for one year [42]. Furthermore, the studies on BCAA supplementation that have been conducted on physically active humans show that a rather large dietary excess of the three BCAAs is well tolerated when consumed in diets containing surfeit amounts of protein. Ingestion of BCAAs in the diet up to 450 mg·kg^−1^ body weight per day, which is a little over three times the estimated average requirement, appears to cause no adverse effects in healthy adults [43].

## 5. Conclusions

In conclusion, this study revealed that chronic BCAA supplementation led to increased performance in rats subjected to a swimming test to exhaustion following moderate-intensity swimming training. The benefits of the diet supplemented with 3.57% BCAA are related to peripheral fatigue but not to central fatigue mechanisms in our experimental conditions. However, the chronic ingestion of a diet supplemented with 4.76% BCAA led to a reduction in performance. 
